# Applications of Federated Large Language Model for Adverse Drug Reactions Prediction: Scoping Review

**DOI:** 10.2196/68291

**Published:** 2025-09-08

**Authors:** David Guo, Kim-Kwang Raymond Choo

**Affiliations:** 1 Department of Information Systems and Cybersecurity, The University of Texas at San Antonio, 1 UTSA Circle, San Antonio, TX, 78249, United States, 1 (210) 458-6300

**Keywords:** adverse drug reactions, distributed systems, federated learning, scoping review, large language model, open-source, fine-tune

## Abstract

**Background:**

Adverse drug reactions (ADR) present significant challenges in health care, where early prevention is vital for effective treatment and patient safety. Traditional supervised learning methods struggle to address heterogeneous health care data due to their unstructured nature, regulatory constraints, and restricted access to sensitive personal identifiable information.

**Objective:**

This review aims to explore the potential of federated learning (FL) combined with natural language processing and large language models (LLMs) to enhance ADR prediction. FL enables decentralized training across client clusters with limited resources, while LLMs effectively process unstructured health care data. By aggregating client-trained models into a global model, FL ensures broader data inclusion while maintaining privacy.

**Methods:**

A scoping review was conducted on peer-reviewed publications retrieved from Google Scholar and Semantic Scholar between 2019 and 2024.

**Results:**

Following the PRISMA (Preferred Reporting Items for Systematic Reviews and Meta-Analyses) protocol, 145 articles from PubMed, arXiv, IEEE, and ACL Anthology met the inclusion criteria. Of these, 12 articles were selected for an in-depth review to examine use cases in ADR prediction. We synthesized ADR data sources on structured and unstructured data types, use cases of FL integrated with natural language processing, and open-source frameworks for ADR identifications and predictions. Special attention is given to unstructured ADR prediction using federated learning with large language models, including development and deployment strategies and evaluation metrics.

**Conclusions:**

Given the recent emergence of LLM, the integration of FL and LLM for ADR prediction remains in its early stage, with limited documented use cases. This review explored the potential applications and highlighted the advancements of federated learning with large language models in health care research, particularly in ADR prediction. Key focus areas include fine-tuning and merging algorithms, fairness and unbiasedness, implementation challenges, and real-world deployment strategies. By synthesizing current insights, this review aims to lay the groundwork for future research in privacy-preserving and scalable ADR prediction systems.

## Introduction

Adverse drug reactions (ADR) are a major concern in the field of medicine, posing serious risks to patient safety. It is estimated that as many as 98,000 people die each year from medical errors that occur in hospitals [1]. This figure surpasses deaths reported from motor vehicle accidents, breast cancer, or AIDS [2,3]. In the United States, ADRs have been reported to the FDA Adverse Event Reporting System (FAERS), which has been maintained by the US Food and Drug Administration (FDA) since 2004. In 2022 alone, over 1.25 million serious adverse events (AEs) were reported, with nearly 175,000 fatalities.

ADR can range from mild symptoms, such as nausea and dizziness, to severe and life-threatening conditions like anaphylaxis and cardiac arrest [4]. As the number of available drugs on the market continues to grow, early identification of potential ADRs is crucial to minimize adverse risks and improve patient safety. Reliable prediction of ADR can play a critical role in preventing harmful medical outcomes. However, health care data are highly regulated due to privacy protection laws such as the Health Insurance Portability and Accountability Act in the United States and the General Data Protection Regulation in Europe. Given the distribution of vast amounts of sensitive health care data across different locations, federated learning (FL) has emerged and proved as a promising solution to address data privacy concerns by enabling the training of machine learning (ML) models on decentralized data sources while maintaining data privacy and security [5-7]. Research on FL in health care, particularly for ADR prediction, is still in its early stages. Most current studies focus on structured outcome prediction. Comparisons between centralized learning and distributed learning, like FL, some research has shown that FL for ADR prediction with electronic health records (EHRs) can achieve higher accuracy than centralized learning approaches [8-10].

A major challenge in health care data usage is that most of the data exist in unstructured and heterogeneous formats that limit its full potential. Recent advancements in natural language processing (NLP) techniques, coupled with enhanced computational power from the latest GPU (Graphics Processing Unit) hardware, have demonstrated the abilities to uncover hidden patterns and relationships in public datasets [11]. One of the most successful applications of NLP is the large language model (LLM), a type of artificial intelligence (AI) model pretrained on vast amounts of text data to perform NLP tasks with high accuracy and adaptability. However, no open-source LLMs have ever been pretrained on public or private ADR datasets, given the high costs and technical barriers. Fine-tuning the open-source LLM on domain-specific data has emerged as a practical approach to gain new domain knowledge on ADR. By learning linguistic patterns and the relationship between inputs and outcomes of ADR training datasets, an adapted LLM can improve its predictive capabilities for ADR in both existing or newly developed drugs [12,13].

Most FL applications have been applied to parameterized, gradient, or transformer-based ML models [11,14,15]. The integration of FL with LLM, known as federated learning with large language model (FedLLM), has gained traction since the emergence of LLMs in 2022. By leveraging FL techniques to train LLMs on distributed data, FedLLM offers several advantages over traditional centralized training procedures [16,17]. This approach inherently enhances data privacy and security by training the model on decentralized data sources. It therefore mitigates the risks associated with centralized data repositories. FedLLM maximizes the potential of LLMs in processing unstructured data without requiring feature engineering, as needed in traditional ML methods. In addition, it enables the inclusion of diverse and heterogeneous ADR data from both public and private sources, leading to more accurate ADR predictions with broader data access. Furthermore, the FedLLM is highly scalable for the integration of multimodal data beyond text and adapts to evolving health care needs. Altogether, FedLLM makes it a promising solution for future ADR prediction and analysis.

This paper focuses on the application of FedLLM for ADR prediction, highlighting its advantages and broader potential applications in the health care industry. We also explore how FedLLM can facilitate collaboration among researchers, institutions, and the health care sector, enabling more comprehensive and dynamic ADR research with innovative technologies. A key benefit of FedLLM applications is their independence from original training data, allowing for real-time and personalized ADR risk assessment based on unstructured individual patient characteristics. In addition, we examine the challenges associated with the implementation of FedLLM in health care and outline future directions for this promising area of research.

This scoping review follows the guidelines of the PRISMA-ScR (Preferred Reporting Items for Systematic Reviews and Meta-Analyses extension for Scoping Reviews) framework [18] (Checklist 1). Our objective is to assess the progress of FedLLM in health care, with a particular focus on the potential for AE or ADR research. By evaluating its effectiveness in handling sensitive, heterogeneous, and unstructured data, we aim to provide a deeper understanding of its applications in ADR identification and prediction. As a result, we hope this review offers valuable insights for various stakeholders, including health care providers, practitioners, and policy makers, into the limitations and challenges associated with using FedLLM beyond ADR prediction. These insights will help inform decision-making regarding its broader application in health care initiatives. The remainder of this paper is structured as follows: The Methods section discusses the review protocol, the Results section outlines the findings on the FedLLM relevant to ADR research, and the Discussion section includes the conclusion followed by future work.

## Methods

### Information Sources and Search Criteria

Our literature search was conducted using 2 search engines, GS and SS, for peer-reviewed articles published since 2019. In both cases, an initial query statement was conducted with keyword combinations as “language model” (“adverse event” OR “adverse drug”) and “federated learning” on GS. The query statement on SS is “language model federated learning adverse events drug reactions prediction.”

### Inclusion and Exclusion Criteria

This study focuses on the review of applications of FedLLM on a broader topic of AEs, in particular ADRs. Specifically, it examines how FedLLM could or has been used to train a language model on public and private data with restricted access within the client storage space. As such, articles regarding the general FL techniques with no applications on ADR or predefined features for structured ADR outcome prediction are excluded. In addition, only articles published in peer-reviewed journals and conference proceedings with PDF available are considered, whereas other types of publications, such as dissertation papers, conference abstracts, book chapters, editorials, and commentaries, were excluded. Also, the articles referenced in this review are limited to publication in English.

## Results

### Data Extraction

A comprehensive search on journal articles was conducted as of July 15, 2024, focusing on publications from 2019 onward. After removing nonrelevant and duplicate articles from the 2 search engines mentioned in the Information Sources and Search Criteria section, only those articles meeting the following inclusion criteria were selected: (1) using unstructured data, (2) incorporating FL, and (3) having full text or PDF copy available.

**Figure 1.**
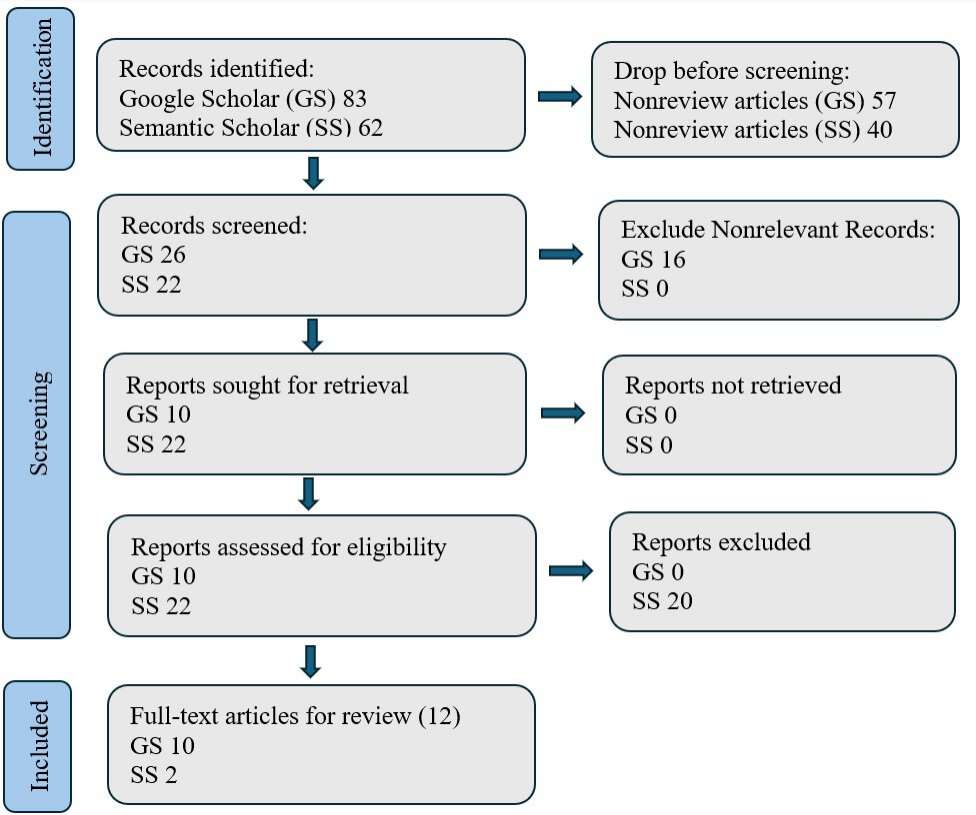
Literature search strategy for FedLLM in predicting adverse drug reactions.

Figure 1 demonstrates the data sources and search flowchart. GS returns a total of 83 articles, including 28 review papers. Together with 62 articles returned from the SS search platform, a total of 145 articles were used as raw inputs. Each full-text paper was reviewed thoroughly to support the quantitative and qualitative synthesis of the data.

### Characteristics of Sources of Evidence

The included articles have a mix of diverse ranges of informatics and biomedical literature, including systematic reviews, meta-analyses, and original research articles sourced from PubMed, arXiv, IEEE, ACL Anthology, and other public databases. To minimize the selection bias, we used a 2-stage screening process involving title and abstract review followed by full-text assessment, ensuring transparent and reproducible study selection.

This study synthesizes findings from 12 key review articles identified through a systematic search of SS and GS. Selected articles focused on ADR-related research with primary emphasis on either language model, FL, or both. We extracted and compared 4 critical dimensions from these reviews: (1) primary research tasks, (2) scope of ADR coverage, (3) methodological approaches focusing on techniques used, and (4) reported limitations or challenges as illustrated in Table 1.

**Table 1.** Comparative summary of review articles.

Review articles	Tasks	ADR^a^ coverage	Techniques used	Limitations or challenges
DeepHealth: review and challenges of artificial intelligence in health informatics (2020) [19]	Imputation methods for missing data Annotation Clinical events Prediction by ML^b^, LR^c^, RF^d^, and NN^e^	Named entity recognition of ADR	Word embedding and unsupervised learning Skip-gram representation CNN^f^ with word2vec DeepCare framework LDA^g^ for topic modeling SDA^h^ for generalizing clinical notes	Accessibility of health data is limited No continuous chronic illness data Unstructured online data Data collection is not consistent
A comprehensive review and application of interpretable deep learning model for ADR prediction (2022) [20]	Query-based search Data integration Data charting Summarization	Pharmacovigilance centers Spontaneous reporting system Drug-ADR association ADR prediction Geographic distribution of ADR research	Disproportionality analysis Fuzzy decision making Temporal association mining ML and DL^i^ NLP^j^	Limited to drug-drug interaction and genetic interaction of drugs Limited to clinical research
A review of federated learning: algorithms, frameworks, and applications (2023) [21]	Joint training Integration of training datasets Prevent data leakage Transfer learning for small dataset	ADR and mortality rate	Vertical FL^k^ Differential privacy Secure aggregation Homomorphic encryption Federated transfer learning	Data leakage during transfer Small data fail to train global model Data share between deep networks Statistical heterogeneity of model Limited overlap of data
Adverse drug event detection using natural language processing: a scoping review of supervised learning methods (2023) [22]	Ensemble method-based ADR extraction Identify ADR in clinic notes Detect ADR from EHR^l^	ADR detection methods by supervised NLP	Supervised NLP	Lack of assessment tool for clinic NLP Limited options to match ontology Lack of data preparation and deployment for NLP
AI in health: state of the art, challenges, and future directions (2019) [23]	NLP for clinic narrative Multiview ensemble classification PubMed tools Interpretable ML CLAMP clinic NLP pipeline	Data mining on FAERS^m^ Drug similarity graph for drug-drug interaction prediction Map drug names and outcomes	Multiview ensemble learning for classification PubMed Phrases, Labs, LitVar Scalable and accurate DL Graph-CNN EHR and Genomics (eMERGE) Topological analysis Model interpretation	NLP algorithms are error-prone Ultrahigh sparsity of EHR data RNN^n^-based models weak on long-term dependencies NLP needs well-defined rules or regular expressions
Causality mining in natural languages using machine and deep learning techniques: a survey (2021) [24]	Mining valuable relational information Automated knowledge extraction Corpus associated Exploring the causality effect	Causality relations of ADR	DL MXNet Caffe, Theano CNTK^o^ Neon DL Gluon	Extract causation knowledge from text Relationship of syntax, semantics, vocabulary, and context in textual data
Handling temporality of clinical events with application to adverse drug event detection in electronic health records: a scoping review (2024) [25]	Data mining Temporal abstraction of EHR Time series classification	Temporality of EHR data for ADE^p^ identification and detection	Temporal abstraction ML prediction Data mining	Limited to ADR on EHR data Limited to classification of multivariate TS^q^ and temporal abstraction features
Machine and cognitive intelligence for human health: systematic review (2022) [26]	Public health surveillance AI^r^ in health care Web-based UI^s^ Ontology development	ADR information extraction with data mining	NLP Web-based semantic system Health care support system Real-time case finding algorithm Topic modeling	Lack of standardized procedure Data quality concern Scalability and generalizability issues Ethical consideration Lack of transparency
Differentially private federated learning: a systematic review (2024) [27]	Differentially private FL for health care applications—classification, topic modeling, and recommender systems	Differential privacy in FL with EHR for ADR prediction	Poisson binomial RDP^t^ LWE^u^ RDP Discrete Gaussian RDP Advanced sequential composition theory Basic sequential composition theory	Limited to a few specific techniques Limited to subset of applications Insufficient discussion of challenges Few concrete case studies
The role of large language models in transforming emergency medicine: scoping review (2024) [28]	Summarize and report the applications of LLMs in EM^v^ by case studies	ADR identification and audits	ChatGPT PaLM^w^ and its variants BERT^x^ and its variants	Lack of discussion on FL with data security concern Lack of coverage on open-source LLMs Lack of comprehensive evaluation on studies
Assessing the performance of large language models in literature screening for pharmacovigilance: a comparative study (2024) [29]	Application of LLMs for literature screening in health care	Systematic monitoring of ADR and the detection of potential safety concerns related to drugs.	Prompt engineering of LLM Fine-tuning LLMs	Less coverage on open-source LLMs Less discussion on the LLM applications
The use of artificial intelligence in pharmacovigilance: a systematic review of the literature (2022) [30]	AI in patient safety and pharmacovigilance	Modeling algorithms for ADR signal identification characterization, assessment, and management	Classification algorithms Text mining for safety narratives	More experimentation studies than concrete case studies Lack of discussion on the integration of social media
This review paper	FL with LLM Performance matrix ADR data sources Implementation pipeline Development and deployment options	Prediction of structured and unstructured ADR outcomes by FedLLM	BERT-based encoder language models BERT-based embedding models Open-source LLMs RAG^y^ Fine-tuning language models Open-source framework	

a ADR: adverse drug reactions.

b ML: machine learning.

c LR: logistic regression.

d RF: random forest.

e NN: neural networks.

f CNN: convolutional neural network.

g LDA: linear discriminant analysis.

h SDA: stack of denoising autoencoders

i DL: deep learning.

j NLP: natural language processing.

k FL: federated learning.

l EHR: electronic health record.

m FAERS: FDA Adverse Event Reporting System.

n RNN: recurrent neural network.

o CNTK: Microsoft Cognitive Toolkit.

p ADE: adverse drug event.

q TS: time series.

r AI: artificial intelligence.

s UI: user interface.

t RDP: Remote Desktop Protocol.

u LWE: Learning With Errors,

v EM: emergency medicine.

w PaLM: Pathways Language Model.

x BERT: Bidirectional Encoder Representations from Transformers.

y RAG: retrieval-augmented generation.

### Language Model for ADR Prediction

Given the nature of unstructured health care data, including patient demographic data, medication administration, and treatment purpose, leveraging the power of language models is a promising approach for ADR prediction. Many current efforts use pretrained Bidirectional Encoder Representations from Transformers (BERT) as the base model to fine-tune on a customized dataset for a downstream task [31]. Through fine-tuning on a large amount of unstructured medical domain data, these models can acquire domain-specific medical knowledge, also known as transfer learning. However, BERT by itself, as an encoder focused on representation learning, is not sufficient to fulfill a prediction task on a defined target. To address this issue, additional decoder layers should be developed on top of the encoder, such as a bidirectional LSTM, can be incorporated on top of BERT to fulfill the prediction task of structured targets. Thus, the BERT framework usually contains 2 steps: pretraining and fine-tuning. The first step is unsupervised training on the unlabeled data, and the second step uses the model parameters from the first step to fine-tune the labeled data, such as binary outcomes ADR (1) and non-ADR (0) [32,33].

Recently, Generative Pre-trained Transformers, a type of LLM represented by ChatGPT from OpenAI, have been pretrained on huge amounts of unannotated and unstructured public data using thousands of high-profile Nvidia GPUs over several months. These models are designed to generate human-like responses in chatbot-style conversations with a strong ability to understand and follow user instructions or prompts [34]. This generative approach expands possibilities for extracting insights and performing complex ADR-related tasks and enables more efficient and adaptive analysis in response to specific queries.

### Summarization of ADR Types

According to the International Conference on Harmonization, ADR is defined as “A response to a drug which is noxious and unintended, and which occurs at doses normally used for prophylaxis, diagnosis, or therapy of disease or the modification of physiologic function” [35]. Based on symptoms, onset time, and nature, ADRs are commonly categorized into 3 primary types. Type 1 reactions can be referred to as augmented reactions, which are dose-dependent and predictable according to the pharmacology of the drug. Type 2 reactions can be referred to as time-dependent reactions. This type of ADE usually shows chronic and delayed effects. Type 3 reactions are unexpected reactions other than the type 1 and type 2, making them idiosyncratic and unpredictable from the perspective of drug pharmacology. Beyond dosage, additional factors can also contribute to the ADR, such as the duration of the reaction and susceptibility factors, including genetic, pathological, drug-to-drug interactions, and other demographic and biological differences. Taking all these factors into consideration will help mitigate any possible ADR and reduce the risk of harm to the patient in clinical practice.

In view of the reported outcomes, ADR can be classified into two classes: (1) unstructured outcome in text, such as the reaction files in FAERS, released quarterly by the FDA, report ADRs using preferred terms defined by the Medical Dictionary for Regulatory Activities [36], and (2) structured outcome summarized in discrete codes, such as the outcome codes in FAERS quarterly released defining discrete outcome in medical code for every primary ID, for example, “DE” for death, “LT” for life-threatening, “HO” for hospitalization—initial or prolonged, “DS” for disability, “CA” for congenital anomaly, “RI” for required intervention to prevent or permanent impairment or damage, and “OT” for serious medical events. Similarly, the AEs are graded by severity from 1 to 5 according to the Common Terminology Criteria for Adverse Events version 5.0 (Table 2).

**Table 2.** Sources of adverse drug reactions data (information accurate as of July 15, 2024).

ADR^a^ data base	Data type	Data sources and number of records
Adverse Drug Event Corpus (Cadec) [37]	Unstructured and structured	AskaPatient, a web-based medical forum where patients can share their experiences with medications, including drug, adverse effect, disease, symptom, finding. The text annotated with ADR is mapped to the corresponding MedDRA^b^ term.
EMA^c^ [38]	Unstructured and structured	The EMA coordinates the European Union pharmacovigilance system and operates services and processes to support pharmacovigilance in the European Union. The database is named the Union Pharmacovigilance Database (UPD).
FDA^d^ Adverse Event Reporting System (FAERS) [39]	Unstructured and structured	FDA’s postmarketing safety surveillance program collects and reports medication errors and product quality complaints resulting in adverse events and release cases on a quarterly basis.
CAERS^e^ [40]	Unstructured and structured	The CAERS contains information about suspected adverse reactions (also known as side effects) since 1965. It is grouped by 26 system organ classes and reported by: (1) consumers and health professionals, who submit reports voluntarily; and (2) manufacturers and distributors (also known as market authorization holders), who are required to submit reports according to the Food and Drugs Act.
DAEN^f^ - medicines[41]	Unstructured and structured	The DAEN - medicines contains information from reports of adverse events (also known as side effects or adverse reactions) received in relation to medicines, vaccines, and biological therapies used in Australia since 1971.
Social Media Mining for Health (SMM4H) [42]	Structured	Ten tasks include English and Spanish posts on Twitter (subsequently rebranded as X), Reddit, and WebMD.
National NLP^g^ Clinical Challenges (n2c2) [43]	Unstructured and structured	shared task on adverse drug events and medication extraction in electronic health records primarily used RNN^h^ consisting of bidirectional LSTM^i^ units.
ADE_Corpus_V2 [44]	Unstructured and structured	This is a dataset that contains ADR data for classification if a sentence is ADE^j^-related (true) or not (false) and relation extraction between ADE and drug.
Common Terminology Criteria for Adverse Events (CTCAE) v5.0 [45]	Unstructured and structured	The NCI^k^ Common Terminology Criteria for Adverse Events is a descriptive terminology which can be used for AE^l^ reporting. A grading (severity) scale is provided for each AE term.

a ADR: adverse drug reactions.

b MedDRA: Medical Dictionary for Regulatory Activities.

c EMA: European Medicines Agency.

d FDA: Food and Drug Administration.

e CAERS: Canada Vigilance adverse reaction online database.

f DAEN: Database of Adverse Event Notifications.

g NLP: natural language processing.

h RNN: recurrent neural network.

i LSTM: long short-term memory.

j ADE: adverse drug event.

k NCI: National Cancer Institute.

l AE: adverse event.

### ADR Benchmark Data

Numerous ADR benchmark data are publicly available from the surveillance practice enforced by regulatory agencies and some independent research institutes. Regulatory agencies, such as the FDA in the United States, the Therapeutic Goods Administration in Australia, the European Medicines Agency in the European Union, and the Canada Vigilance Program in Canada, collect volunteer reports of adverse drug side effects, investigate the reports, and issue safety alerts if a drug is suspected of causing an AE. A summary of publicly available ADR benchmark data is presented in Table 2.

### Evaluation Matrices on Prediction Accuracy

For structured ADR outcome evaluation, metrics such as *F*_1_-score, recall, and precision are commonly applied. However, given the predominantly unstructured nature of health care data, this review focuses on evaluating unstructured prediction outcomes leveraging the power of NLP technologies. The predicted outcome will emulate the responses by different individual professionals or nonprofessionals to a specific ADR event with different yet semantically similar texts. The core evaluation of NLP-based ADR prediction involves either leveraging another language model with domain knowledge to evaluate the performance of ADR prediction or incorporating a human-in-the-loop approach, where health care professionals review and validate the predictions. A summary of their strengths and limitations of each evaluation approach is provided in Table 3.

**Table 3.** Evaluation metrics on unstructured predictions.

Matrix	Description	Strength	Limitation	Equation
G-Eval [46]	This metric uses LLMs^a^ with chain-of-thoughts (CoT) to evaluate LLM outputs based on any custom criteria	It is easy to compute and does not require any additional data.	It may not capture the quality of the generated instructions beyond the accuracy of the predicted ADR^b^ outcome	$Score=\sum_{i=0}^{n}p\left({\text{s}}_{\text{i}}\right)*{\text{s}}_{\text{i}}$ where Si_i_ are the predefined scores, and p(Si_i_) are calculated probabilities by the LLM
BLEU_N [47]	This metric measures the n-gram precision based on the similarity between the generated text and the ground truth instructions	It can capture the quality of the generated text beyond the accuracy of the predicted text	It may not be suitable for evaluating the accuracy of the predicted adverse drug reaction as it rewards fluent but incorrect text generation	$p_{n}=\frac{\sum_{c}\sum_{n-gram}Count\left(n-gram\right)}{\sum_{c^{\prime }}\sum_{n-gram^{\prime }}Count\left(n-gram^{\prime }\right)}$ where C denotes the reference count, C’ denotes the reference count, n-gram denotes the clip count
ROUGE_N [48]	This metric measures the n-gram recall between candidate and reference set based on the overlap between the generated text and the ground truth instructions	It can capture the quality of the generated text beyond the accuracy of the predicted text	It may not be suitable for evaluating the accuracy of the predicted text as it rewards overlapping but incorrect text generation	$R_{n}=\frac{\sum_{Se}\sum_{n-gram}Count_{match}\left(gram_{n}\right)}{\sum_{Se}\sum_{n-gram}Count\left(gram_{n}\right)}$ Where Se denotes reference summary, Countmatch_match_(gramn_n_) denotes the maximum number of n-grams co-occurring in candidate and reference summaries
BERTScore [49]	This metric measures a similarity score for each token in the candidate sentence with each token in the reference sentence. Instead of focusing on exact matches, token similarity between the sentences is computed using BERT^c^ contextual embeddings	It can capture the semantic similarity between the generated text and observed text	BERT model is not trained on ADR data; thus, the BERT embeddings may not fully represent the tokens from ADR domain	$R_{Bert}=\frac{1}{|x|}\sum_{x_{i}}\frac{max}{{\hat{x}}_{i}}x_{i}^{T}{\hat{x}}_{j}$, $P_{Bert}=\frac{1}{|\hat{x}|}\sum_{{\hat{x}}_{i}}\frac{max}{{\hat{x}}_{i}}x_{i}^{T}{\hat{x}}_{j}$ $F_{Bert}=2\frac{P_{Bert}*R_{Bert}}{P_{Bert}+R_{Bert}}$ Where RBert_Bert_ denotes the recall of tokens, PBert_Bert_ denotes the precision of tokens, *FBert _Bert_* denotes the *F*_1_score
SEMScore [50]	This metric computes the semantic textual similarity between embeddings of model and target. It consists of two steps, (1) it embeds model and target responses separately using the chosen sentence transformer and (2) it computes the cosine similarity of the respective embeddings as the value of SEMScore	It can capture the semantic similarity between the generated text and observed text based on the cosine similarity of embeddings	Like the BERT score, it also depends on the BERT model with domain knowledge	$cos\left(\theta \right)=\frac{A.B}{\left|\left|A\right|\right|\vee \left|B\right|\vee }=$ $\frac{\sum_{i=1}^{n}A_{i}B_{i}}{\sqrt{\sum_{i=1}^{n}A_{i}^{2}}\sqrt{\sum_{i=1}^{n}B_{i}^{2}}}$ Where A denotes the embedding vector of predicted texts, B denotes the embedding of embedding of observed texts
ANLS*[51]	This metric computes the Average Normalized Levenshtein Similarity (ANLS) with penalty on unanswerable questions for a wide variety of tasks, including information extraction and classification tasks	It is a general metric applicable for a wide variety of tasks with varied output formats, either structured or unstructured	It can only deal with strings and lists but not dictionaries or any combination of types	ANLS*(g, p) = $\frac{s\left(g,p\right)}{l\left(g,p\right)}$ Where, g denotes ground truth, p denotes prediction, s is the score between the ground truth and the prediction, and l is the size of the trees g and p such that ANLS*(g, p) ∈ [0, 1]

a LLM: large language model.

b ADR: adverse drug reactions.

c BERT: Bidirectional Encoder Representations from Transformers.

### FL and LLM for ADR Research

The research and application of FL and LLM for ADR have demonstrated promising results in enhancing the accuracy of ADR detection and summarization. For instance, FL has been applied to ADR detection tasks by leveraging distributed data from multiple sources, such as EHR and claims data. By federating the learning process, FL can improve the accuracy of supervised ADR detection without compromising data privacy and security [52]. In addition, the integration of BERT-based transfer learning with unique molecular embeddings allows for the development of multimodal models that effectively combine language understanding and molecular property prediction, thereby enhancing adverse drug event classification [53]. Recently, LLMs have been used to summarize and extract ADR-related text, such as clinical notes and medical articles. By using pretrained language models to encode text, LLMs can capture the semantic meaning of ADR-related phrases and generate accurate summaries. A multitask learning approach that combined question summarization and recognizing question entailment was used to improve the performance of pretrained language models in ADR summarization [54].

Given the fact that most distributed ADR data are imbalanced, data augmentation–based methods have been proposed to improve the performance of LLMs in ADR summarization. These approaches generate additional training data inspired by a data augmentation method in NLP that generates synthetic sentences for training by replacing some words with syntactically and semantically similar words [55].

Zero-shot–based methods can adapt LLMs to ADR-related tasks without the need for additional labeled training data. These methods typically use transfer learning to leverage pretrained language models and fine-tune them for ADR-related tasks. This technique is particularly useful for detecting rare or unexpected ADR, as well as for identifying potential drug-drug interactions that may not be captured by traditional ADR detection methods [56].

## Discussion

### Strategies for FedLLM on ADR

Most reported use cases of FL with language models leverage the knowledge acquired by the pretrained model on a large dataset and apply it to a domain-specific task with a smaller dataset through transfer learning, represented by a customized fine-tuning process [57-59]. This process involves taking a pretrained model and further adapting it to a new task by adjusting its parameters. To facilitate the fine-tuning of language models on client data while reducing computational demands on limited client infrastructure, several strategies should be considered: (1) configure a high dropout rate on client model; (2) reduce model precision through quantization, such as using 4-bit instead of 16-bit precision; (3) segment each parameter matrix of the large model into submatrices with varying reduction ratios; and (4) transfer only distilled-federated knowledge back to server, for example, at the end of the training process on client data, only the student model from a teacher-student model framework is shared to minimize communication costs.

Before fine-tuning, it is crucial to optimize the process to compromise the low-profile computation capability of client facilities to achieve the best training performance, including a systematic evaluation of options among inclusive FL, exclusive FL, and hetero FL, which is necessary to achieve fairness across client and global models [60]. For example, using the distilled model can allow more client data to be included in the training process. Taking Named Entity Recognition (NER) for ADR as an example, adoption of distillation models, such as DistilBERT to fine-tune ADR data, achieve even better performance than fine-tuned LLMs xlm-RoBERTa and GPT-Neo-125M in all 4 metrics, that is, accuracy, precision, recall, and *F*_1_-score [61]. In an evaluation study for adverse drug event extraction, PubMedBERT, distilled from GPT-3.5 (OpenAI) with merely 0.1% of model size, outperformed its parent model by over 6 absolute points in *F*_1_-score, and a distilled model from GPT-4 achieved an improvement of 5 absolute points [62].

### Fine-Tuning Framework for ADR FedLLM

There are 2 types of LLMs, closed-source and open-source. The closed-source models like ChatGPT (OpenAI) and Gemini (Google) are pretrained on public data without ADR domain knowledge. The weights of models are not publicly available; we therefore discuss the fine-tuning strategies limited to open-source pretrained models shared on the Hugging Face website with Apache-2.0 license, such as Llama-3, Gemma, Mistral/Mixtral, Falcon, DeepSeek, and Qwen [63]. The fine-tuning process involves the preparation of ADR instruction data as new training data for a domain-specific task, in this case, the prediction of structured and unstructured ADR outcomes. A widespread practice of fine-tuning LLM usually applies low-rank adaptation, which freezes the weights of the pretrained model and injects trainable rank decomposition matrices into each layer of the LLM transformer architecture. This process significantly reduces the number of trainable parameters down to less than 1% of the original parameters, given the selection of rank [64,65].

The implementation of FedLLM can use the frameworks and libraries that support FedLLM, such as TensorFlow Federated (a framework for implementing FL algorithms using TensorFlow), PySyft for PyTorch developed by OpenMined, Federated AI Technology Enabler (an FL framework by WeBank), and Federated Learning Application Runtime Environment by Nvidia [66]. In all cases, the general workflow includes (1) declaring LLM and defining FL parameters, (2) training client data on client infrastructure with shared parameters from global model and returning the model weights to server which will be used to update the global model with aggregated weight (this process could be a human-involved process), and (3) model evaluation on validation dataset and deployment of global model for low latency and scalable inference.

After a client model has been fine-tuned, only model weights or gradients instead of private training data will be shared with the server and merged with the global model into an updated new model. The straightforward method to merge client model parameters is to average the weights of the client and global models [67]. Other advanced merging processes include Spherical Linear Interpolation, which takes weight of each model into consideration [68], Drop and Rescale, which can effectively eliminate 90% or even 99% delta parameters of multiple supervised fine-tuned models [69], and the model stock approach, which approximates a center-close weight using only 2 fine-tuned models [70].

### FL on Embedding Models

Ideally, a FedLLM pretrained or fine-tuned ADR data would be preferable for direct ADR prediction. As of the time of this review, none of the open-source LLMs have been pretrained for ADR use cases. Alternatively, for retrieval-augmented generation (RAG)–based ADR prediction, any open-source LLM without ADR domain knowledge can indirectly predict ADR outcome by leveraging the retrieved evidence from similarity search between the prompt embedding and prepopulated embeddings in the vector database. This approach allows any LLM to function as a summarization engine. Compared with the LLM, a fine-tuning embedding model is more manageable, as a transformer-based embedding model is typically much smaller than an LLM by an order of 10 to 100 magnitudes. This reduced model size translates to less computation resource requirement for fine-tuning an embedding model on a small set of private ADR data, thereby gaining distributed ADR domain knowledge.

With an embedding model, the distributed ADR-specific terms and concepts, such as diseases, symptoms, and treatments, can be transformed into dense vectors, also known for embeddings that capture the semantic relationships between medical entities, allowing AI models to work within a mathematical framework [31,71,72]. Since most embedding models are rooted in the BERT model, the length of embeddings is typically 512 or more; the longer the embedding, the better ADR information will be represented. These embeddings can be indexed and hosted in a vector database for rapid and accurate content search within a RAG pipeline. Most open-source vector database options use Hierarchical Navigable Small World as a vector index type. This graph-based algorithm efficiently identifies nearest neighbors among large, multidimensional vectors without requiring a full scan of the entire vector database [73].

Like the fine-tuning FedLLM, FL on the ADR embedding model can also be accomplished by fine-tuning a selected foundation embedding model on client data within resource-constrained infrastructure with customized settings. Some embedding foundation models are not BERT-based models, such as Word2Vec on the Hugging Face website, which is trained on biomedical text to learn the vector representations of medical terms based on their co-occurrence patterns in large text corpora like PubMed articles. Most foundation embedding models, however, are BERT-based. For example, BioBERT is specifically fine-tuned on biomedical text to provide contextual embeddings for biomedical entities [74]. It can enhance performance on tasks like NER and biomedical question answering. ClinicalBERT is pretrained on clinical notes and EHR, capturing nuances specific to medical contexts [75]. It supports varied tasks such as clinical entity recognition, medical coding, and patient phenotype extraction. All these embedding models capture entire sentences, paragraphs, or even longer texts. This distinction capability allows any LLMs to understand medical terminology at a contextually rich level.

### Implementation of FedLLM for ADR Prediction

The implementation of FedLLM inference for ADR prediction is usually not a topic in academic research. Its importance should never be underestimated. It is the last mile of a FedLLM modeling effort to connect to the end users. It requires a GPU-supported backend to meet the significant computational demands of FedLLM processing. GPUs provide the parallel processing capabilities necessary for handling complex model architectures, enabling faster inference and efficient handling of large-scale datasets. This is particularly critical in both the FL process and afterward model inference, where distributed data processing and model inference must be performed seamlessly to ensure scalability, accuracy, and real-time responsiveness in ADR prediction tasks.

The interaction between the end user and FedLLM relies on prompt engineering techniques, which help transform a user query into a FedLLM-compatible prompt by incorporating the query as part of the context. This process is facilitated through a user interface (UI) serving as the front end, typically designed as a chatbot. Such a design enhances user engagement, particularly within healthcare settings, where individualized, real-time, and professional responses are essential. During inference, FedLLM can be hosted either on a third-party cloud or an internal on-premises cloud. Opting for a third-party cloud implementation solution could be more practical for the institute when addressing technical debt. In fact, integration of prompt engineering techniques with other settings has demonstrated improved effectiveness in detecting AE or ADR [29].

There are 2 ways to use FedLLM as the back end for ADR prediction. The first method involves directly submitting the prompt to a fine-tuned FedLLM without a vector database search. This approach can significantly reduce response latency. However, its limitation is clear—newly acquired ADR information cannot be immediately incorporated into the ADR prediction process after FedLLM has been fine-tuned. In addition, the cost of fine-tuning is nontrivial. A more flexible approach involves integrating the FedLLM into the RAG pipeline. In this setup, the search engine will calculate the similarity of the embedding of the user input against a prepopulated vector database to identify the top K relevant ADR outcomes, which are then processed into a customized prompt before being submitted to FedLLM for ADR prediction. For example, the telehealth system for video-based consultations and remote monitoring can help reduce the workload of healthcare providers while improving patient outcomes [76].

Several open-source Python-based tools facilitate lightweight UI development. With Streamlit being a notable example, this open-source framework enables data scientists and AI and ML engineers to create and deploy interactive, chatbot-style web applications [77]. It also allows the integration of chatbots powered by LLM through open-source frameworks, such as Llama Index [78] and LangChain [79].

To facilitate the development and deployment of UI in pilot tests (also known as the user acceptance test) and in production, options include web-based UI deployment and container-based UI deployment. However, both methods should be implemented under tight access control and physician supervision to prevent misuse of the tool. Running a lightweight pilot test is designed to evaluate the model’s performance through small-scale user acceptance testing. To achieve this goal, both web-based and container-based deployments present practical solutions. Depending on the readiness of the client facility for Kubernetes-based continuous integration and continuous delivery or deployment pipeline, the continuous integration process can ensure the incremental code changes are made frequently and reliably. This setup allows Python development code to be quickly and seamlessly deployed as a part of the continuous delivery or deployment process directly from development to production. Alternatively, packaging the entire development environment into a portal image for local container-based deployment is becoming popular due to its portability and scalability, either using a daemon-dependent Docker engine [80] or a daemon-independent Podman engine [81].

### Challenges and Limitations of FedLLM in ADR Research

Numerous limitations of FedLLM for ADR research and application have been highlighted in the selected articles. The primary limitation is the lack of fine-grained control over the data collection process. While open-source scraping and crowdsourcing are 2 common methods for collecting large textual datasets, both methods face challenges related to observational bias, scalability, and the ability to exert fine control over the data collection process. Although the crowd-sourcing platforms can be more expensive than open-source alternatives, they do provide greater control over the data collection process [82]. Therefore, the main limitation of FedLLM for ADR research and application is the need for more controlled and diverse data sources to improve the accuracy and generalizability of the models.

The inherent server-client coupling of FL systems also increases the complexity of the overall architecture and compromises the integrity of client environments. In addition, the limited reusability of FL models may lead to suboptimal performance in some specific scenarios. Most FL systems are nonpublic, meaning that the data used for training are not available for public scrutiny. The lack of transparency restricts the reproducibility of results and limits the applicability of the model in certain situations. In addition, the performance of the global model can be sensitive to the number of clients participating in the federated averaging process, and a sudden drop in performance in some scenarios may be attributed to smaller data partitions and inconsistencies among the participating models [83].

### Security of FedLLM

FedLLM algorithms, combined with security and privacy techniques, enable federated training. However, posttraining processes of FedLLM exhibit varied vulnerabilities, including but not limited to adversarial attacks, model inversion, backdoor, and data extraction. Inversion attacks aim to reverse engineer and reconstruct the sensitive input data from model output, such as personally identifiable information [84]. Gradient inversion attack poses an emerging threat by attempting to recreate original data from the model gradient, highlighting potential security and privacy breaches in FL. It is important to note that some models may memorize some specific data points rather than general patterns due to the possible overfitting on a small training dataset [85]. In contrast, adversarial attacks seek to deceive models by injecting carefully curated adversarial samples designed to induce incorrect predictions or unexpected behaviors [86]. This poses substantial risks to critical applications, such as ADR prediction, which has critical consequences such as injuries and fatalities. Mitigation strategies to defend against adversarial attacks require joint approaches, including having critical consequences such as injuries and fatalities. Mitigation strategies to defend against adversarial attacks require joint approaches, including (1) adversarial training with adversarial samples to enhance the model’s resilience against adversarial perturbations, (2) input validation before training can prevent potential adversarial modification by identifying and rejecting suspicious data from inputs, and (3) gradient masking which limits the information available to adversarial attacks, making it more challenging for attackers to deceive the model.

Various strategies have been applied to enhance model security against potential threats, including corpus curation, application of a penalty on the training loss, instruction-based fine-tuning, classification of sensitive data samples, and constrained direct preference optimization. Among these efforts, instruction fine-tuning with representative examples proved to be a promising solution [87].

### Interpretability of FedLLM

Given the heterogeneous nature of client data and the complexity of client models due to diverse client infrastructure, the explanation and interpretability of FedLLM remain complex topics. However, various techniques and tools can enhance the interpretability of FedLLM. Transformer architecture, the foundation of the most modern LLMs, uses an attention mechanism to weigh the importance of different parts of the input sequence when generating outputs, a process known as self-attention. Beyond self-attention, multihead attention enhances the model’s ability to capture diverse contextual information by simultaneously attending to different parts of the input sequence. It achieves this by performing multiple parallel self-attention operations, each with its own set of learned query, key, and value transformations [88].

Understanding how attention mechanisms operate and interpret their output can be challenging. Techniques like attention attribution help identify the contributions of individual tokens to model predictions, thereby improving explainability. Methods for visualizing attention weights and interpreting their significance have been developed to enhance the interpretability of multihead self-attention in transformer-based language representation models [89].

In contrast to the fixed relationship established by word embedding regardless of its context within a sentence or document, attention mechanism solves this challenge by enabling models to selectively focus on relevant parts of input sequences, thereby incorporating context sensitivity into the representation learning process in tasks requiring a nuanced understanding of language, especially when words carry different contextual meanings [90]. Therefore, a fine-tuned LLM with ADR domain knowledge can be the answer to the explainable AI when trained on sufficient public and private ADR data.

With RAG technologies, the prediction of ADR outcome becomes a joint effort of FedLLM and RAG, initiated by a prompt query on the ADR vector database. With no exception, all ADR data used to build the vector database should be free of personally identifiable information, which can be achieved through NER and information masking techniques. The top K (K≥1)-relevant ADR cases returned from a similar search will be used as context and incorporated into the prompt for ADR prediction by FedLLM. In this model, the fine-tuned FedLLM does not work alone to predict the ADR outcome; rather, it serves primarily as an information summarization tool for ADR prediction. The retrieved ADR cases are not only used for ADR prediction but also presented to the user as source citations, making the decision-making process of FedLLM more transparent and interpretable.

### Computation Costs and Environmental Impact

Unlike the pretraining LLM, which requires orders of magnitude more data and computational power, fine-tuning an ADR LLM uses much less data and resources while enhancing performance on a targeted downstream task. However, due to data privacy concerns, fine-tuning LLM still requires an on-site training process with access to high-quality and low-bias data, as well as technical readiness, construction of GPU infrastructure, and energy consumption. With the emergence of AI regulations, such as the EU AI Act [91], requiring simple data lineage, minimizing low data bias, and enhancing energy efficiency have become a priority for everyone offering AI services. This can be a major concern for organizations that are looking to reduce their environmental impact and improve the compliance and sustainability of their operations.

Running inference on LLM for ADR prediction also requires an environment with a GPU or TPU (Tensor Processing Unit) to minimize response latency. It is not a cost-efficient way to set up a private inference infrastructure due to its high-maintenance cost and low usage of GPU, which typically operate at only 20%‐25% of GPU memory, which is in use at any given time on average [92]. Currently, cloud computing services for enterprise LLM inference are offered by providers such as Google Vertex AI, Amazon AWS Bedrock, Nvidia AI Inference platform, and Snowflake Cortex. All these services operate as Infrastructure as a Service, providing centralized computation, data storage, and network resources on a pay-as-you-go basis. Allowing customers to pay for the service they use by token counts.

### Bias and Fairness

The performance of FL algorithms with LLM has been evaluated across various ADR-related tasks, including NER and Relation Extraction [93,94]. However, both open-source and closed-source foundation LLMs are typically pretrained on public data with no specific focus on ADR. Since the weights of closed-source LLMs are not publicly accessible, only open-source LLMs can be used as the foundation model. Most open-source foundation models have been trained to reduce bias before being released for public use. It is important to note that FedLLM may be vulnerable to undesired biases present in the fine-tuning data distributed across clients, which can lead to unfair outcomes, particularly given the fact that the training data should be non–independent and identically distributed. These biases may come from demographic biases, data collection biases, and data sampling biases. To mitigate these issues, researchers should take extra steps in data preprocessing to remove biases and ensure that it is representative of the target population. Regularization techniques, such as dropout or weight decay, can help reduce the impact of biased features on the model’s predictions.

The fairness of FedLLM involves ensuring the model responses do not disadvantage any group and that it does not generate harmful or discriminatory outputs to the end user. To achieve this goal, researchers can design and develop optimized algorithms that incorporate fairness metrics, such as demographic parity or equalized odds regulation, along with fairness-aware gradient descent, to promote fairness and reduce bias [23,95]. In addition, inclusion of adversarial examples that simulate the biases in the fine-tuning data can help the model learn to recognize and correct these biases [96].

### Conclusion and Future Work

Unlike the centralized model training process that brings the client data to code in the server, FL systems adopt a distinct approach by bringing the server code to the client data. This strategy minimizes data movement, thereby preserving data privacy. FL has been widely used for classification tasks on structured health care outcomes. However, the complexity of the overall architecture and concerns about the integrity of client data remain a challenge since most FL use cases are based on nonpublic data, which often lack reproducibility. Therefore, leveraging the FedLLM for synthetic health care data generation will provide a new source to meet a growing demand for unbiased training data [97].

With the prevalence of LLM, which can seamlessly process unstructured data without the need for additional data preprocessing. This approach will make it easy to leverage distributed private data in heterogeneous formats that were previously difficult to use. With fine-grained control over the data collection and preparation process of high-quality training data, more private and public health care data will be accessible for FedLLM fine-tuning tasks.

Furthermore, health care data extends beyond textual information. Medical image data, such as computed tomography and magnetic resonance imaging, have significantly increased the volume and complexity of patient information available for multimodal LLM fine-tuning [98]. A key limitation of FedLLM for ADR research and application is the need for more controlled and diverse data sources to improve the prediction accuracy and generalizability. The latest multimodal open-source LLMs with vision abilities, such as Llama 3.2 vision from Meta and MVLM from Nvidia, are poised to address this demand. Meanwhile, as computational power becomes more affordable and the size of foundational LLMs decreases without sacrificing performance, it is anticipated that FedLLM will gain significant traction in health care applications, particularly in ADR research.

## Supplementary material

10.2196/68291 Checklist 1 PRISMA-ScR checklist.

## Abbreviations

ADR: adverse drug reactions
AE: adverse event
AI: artificial intelligence
BERT: Bidirectional Encoder Representations from Transformers
EHR: electronic health record
FAERS: FDA Adverse Event Reporting System
FDA: Food and Drug Administration
FedLLM: federated learning with large language model
FL: federated learning
GPU: Graphics Processing Unit
LLM: large language model
ML: machine learning
NER: Named Entity Recognition
NLP: natural language processing
PRISMA: Preferred Reporting Items for Systematic Reviews and Meta-Analyses
PRISMA-ScR: Preferred Reporting Items for Systematic Reviews and Meta-Analyses extension for Scoping Reviews
RAG: retrieval-augmented generation
UI: user interface
